# Anesthesia in a Patient with Potocki–Lupski Syndrome

**DOI:** 10.1155/2021/3313904

**Published:** 2021-12-04

**Authors:** Songhyun Kim, Yunhee Lim, In-Jung Jun, Byunghoon Yoo, Kye-Min Kim

**Affiliations:** Department of Anesthesiology and Pain Medicine, Inje University Sanggye Paik Hospital, Seoul, Republic of Korea

## Abstract

**Introduction:**

Potocki–Lupski syndrome (PTLS) is a rare disease caused by the duplication of a small segment of chromosome 17 (17p11.2). The clinical presentation of this syndrome is quite variable and includes hypotonia, failure to thrive, oropharyngeal dysphagia, developmental delay, and behavioral abnormalities. In addition, congenital heart disease, sleep apnea, and mildly dysmorphic features are common and should be considered during anesthetic management. However, because of the rarity and newness of the syndrome, there are few reports on the anesthetic care of patients with PTLS. *Case Report*. We report a case of a 4-year-old girl diagnosed with this syndrome who underwent general anesthesia for exotropia surgery. The patient exhibited micrognathia; a mild decrease in muscle tone; and a developmental delay in motor, speech, and cognition. She had a history of swallowing incoordination and gastroesophageal reflux. No abnormalities were found on a preoperative echocardiography. A videolaryngoscope was used for tracheal intubation, and the state of neuromuscular blockade was monitored in addition to standard monitoring. Anesthesia was maintained with sevoflurane and remifentanil. The patient recovered without any adverse events.

**Conclusion:**

As PTLS patients may have several malformations, preanesthetic evaluation is important. Preoperative echocardiography and cardiologic consultations are required. It is desirable to prepare for the risk of difficult airway and pulmonary aspiration. Postoperatively, close monitoring is needed to prevent airway compromise.

## 1. Introduction

Potocki–Lupski syndrome (PTLS) is a rare chromosomal disease with an estimated incidence of 1/25,000 [1]. PTLS, also known as duplication 17p11.2 syndrome or trisomy 17p11.2, is diagnosed by detecting a heterozygous microduplication at band 11.2 on the short arm of chromosome 17, which encompasses the retinoic acid inducible 1 gene (*RAI1*). The size of the duplicated region varies; however, a common (∼3.7 Mb) duplication is found in approximately two-thirds of patients [2].

Since the molecular mechanism and phenotypes of PTLS were reported only in 2000 [3], the detailed characteristics were identified only recently [2, 4–6]. The typical features of PTLS in infancy include hypotonia with poor oral feeding and a failure to thrive. In childhood, global developmental delay, language impairment, intellectual disability, behavioral abnormalities, and autistic features are commonly observed. In addition, oropharyngeal dysphagia, low muscle tone, and sleep-disordered breathing may also occur. The severity of dysmorphism is mild [2]. It is noteworthy that cardiovascular anomalies and electrocardiographic abnormalities are found in approximately 40% of PTLS patients [4].

As the pathologic presentation of PTLS may affect anesthetic management, precautions are needed in anesthetizing patients with PTLS. However, because of the rarity and newness of the syndrome, there are few reports related to anesthesia in patients with this syndrome.

Here, we report the case of a 4-year-old girl with PTLS who underwent general anesthesia for exotropia surgery and discuss the anesthetic considerations.

## 2. Case Presentation

Written informed consent was obtained from the patient's mother for publication of this case. A 4-year-old girl diagnosed with PTLS was scheduled to undergo exotropia surgery under general anesthesia. She was born by cesarean section on the 4th day of the 38th week of gestation and weighed 3020 g. Immediately after birth, she had a heart murmur and intermittent desaturation with cyanosis; she was, thus, transferred to our hospital for intensive care. She received oxygen therapy and nutritional support due to poor feeding and lack of weight gain. At that time, micrognathia and pectus carinatum were observed, and a small atrial septal defect (ASD) was found on echocardiography. However, follow-up echocardiography performed after 6 months showed normal findings without ASD. During infancy, swallowing incoordination and gastroesophageal reflux were observed; however, they improved afterwards. Around 18 months of age, a developmental delay in motor, speech, and cognition and a reduction in the muscle tone were observed. Peripheral blood chromosome analysis revealed a duplication of approximately 3.8 Mb at chromosome 17p11.2, and PTLS was diagnosed. Since then, she has been receiving rehabilitation therapy, such as occupational, speech, and cognitive therapy. In addition, the cardiology outpatient follow-up was continued.

At the age of 2 years, she was diagnosed with exotropia. When she was 4 years old, she was admitted to hospital for exotropia surgery. The patient weighed 17.9 kg and was 108 cm tall. Since she had micrognathia and a history of swallowing incoordination and gastroesophageal reflux, the possibility of a difficult airway and the perioperative risk of pulmonary aspiration were considered. Laboratory tests, electrocardiography (ECG), and echocardiography performed before surgery showed normal findings. Atropine (0.25 mg) was administered intramuscularly as a premedication. General anesthesia was performed after obtaining informed consent for anesthesia from the patient's mother. ECG, noninvasive blood pressure, pulse oxygen saturation (SpO_2_), esophageal temperature, and a nerve stimulator were used for monitoring during anesthesia. Before anesthetic induction, blood pressure, heart rate, and SpO_2_ were 120/70 mmHg, 114/min, and 98%, respectively. A nerve stimulator (Fisher and Paykel Healthcare, New Zealand) was attached to the left wrist area along the path of the ulnar nerve to monitor the train of four (TOF) counts or ratios. After denitrogenation with 100% oxygen for 5 min, 100 mg of thiopental was injected intravenously. After the loss of consciousness, mask ventilation was performed with sevoflurane in oxygen and 12 mg of rocuronium was injected intravenously. Mask ventilation was easy. Tracheal intubation was performed using a videolaryngoscope (McGRATH™ MAC blade size 2, USA) and a cuffed endotracheal tube (internal diameter, 4.5 mm). In a videolaryngoscopic view, the Cormack–Lehane grade was assessed to be 1. General anesthesia was maintained with sevoflurane and remifentanil. No additional rocuronium was administered during the anesthesia. The duration of the surgery was 45 minutes. At the end of the surgery, the TOF counts were 2. For the reversal of neuromuscular blockade, neostigmine (0.75 mg) and glycopyrrolate (0.1 mg) were administered intravenously. Extubation was performed after the confirmation of adequate recovery of spontaneous ventilation, awakening, and a TOF ratio of 100%.

Upon arrival in the postanesthesia care unit (PACU), the Aldrete recovery score was 10 and the SpO_2_ level was 100%. Oxygen (5 L/min) was administered to the patient via a facial mask with a reservoir bag. In the PACU, the patient did not show any respiratory complications, such as apnea, aspiration, or upper airway obstruction. After staying in the PACU for 20 min, she was transferred to the ward and discharged the next day.

## 3. Discussion

Although the case reports and reviews on PTLS have been published until recently [6–8], there are few reports on the anesthetic care of patients with PTLS [9, 10]; therefore, this syndrome is very unfamiliar to anesthesiologists.

In our case, a 4-year-old girl diagnosed with PTLS showed micrognathia, a mild decrease in the muscle tone, neurodevelopmental delay, and a history of swallowing incoordination and gastroesophageal reflux. However, the clinical manifestations of PTLS are diverse. The typical features observed in most patients are hypotonia, poor feeding in infancy, global developmental delay including language/intelligence impairment, and behavioral abnormalities, ranging from interaction impairment to autism spectrum disorder [2, 6, 11]. In addition, cardiovascular anomalies, electrocardiographic abnormalities, sleep-disordered breathing (central and/or obstructive sleep apnea), and mildly dysmorphic facial features (triangular shaped face, micrognathia, downslanting palpebral fissures, and high arched palate) are frequently present. Electroencephalographic abnormalities, seizure, oropharyngeal dysphagia, gastroesophageal reflux, type 1 Arnold–Chiari malformation, and renal anomalies may also be present [1, 2, 4, 5, 7, 8, 11]. The phenotype of PTLS is so variable that the severity varies from the case of limited social capability due to the developmental disability to the case of almost normal life [6].

The clinical characteristics described above should be considered in the anesthetic management of patients with PTLS. In particular, the presence of cardiovascular involvement, sleep apnea, hypotonia, oropharyngeal dysphagia, swallowing difficulty, and micrognathia should be noted.

In our patient, no cardiac abnormalities were found on preoperative echocardiography. However, in a study by Jefferies et al., 40% of patients with PTLS had cardiovascular involvement, such as structural abnormalities or conduction disorders [4]. Aortic dilation was the most common complication (20% of the patients). Patent foramen ovale, atrial septal defect, ventricular septal defect, and the bicuspid aortic valve were observed in 16%, 12%, 8%, and 8% of the patients, respectively. Serious anomalies, such as a hypoplastic left heart, have also been reported [12]. Therefore, echocardiographic examination for the evaluation of the heart and aortic root is required at the time of the diagnosis of PTLS. Although no abnormalities were found in the echocardiogram, echocardiography should be reevaluated every 2-3 years until adolescence and then every 4-5 years [1]. Considering the high prevalence and possible progression of cardiovascular abnormalities, preanesthetic evaluation of PTLS patients should include echocardiography and cardiologic consultation in addition to electrocardiography.

As the patients with PTLS may have facial dysmorphism, such as micrognathia and high arched palate, anesthesiologists should be prepared for the possibility of a difficult airway. Video laryngoscopy and supraglottic airways may be useful in such cases. At the same time, considering the frequently accompanied oropharyngeal dysphagia and gastroesophageal reflux, the risk of pulmonary aspiration should be considered during perioperative care. Intraoperative monitoring of the neuromuscular blockade is desirable. Since the presence of hypotonia, swallowing incoordination, and sleep apnea can be related to a compromised airway, the complete reversal of neuromuscular blockade at the end of the surgery is recommended. In addition, close monitoring is required during the postoperative period to prevent respiratory complications, such as an upper airway obstruction. Another consideration in the anesthetic management of patients with hypotonia is the susceptibility to malignant hyperthermia (MH). Until now, there is no evidence for an association of PTLS and MH susceptibility. In a previous case report, Urbón et al. used intravenous anesthetics rather than volatile anesthetics regarding possible MH susceptibility, although the relationship between this disease and MH was not clear [9]. Hypotonia in PTLS is of central origin, and since there was no medical or family history related to MH susceptibility in this patient, we considered that the risk of MH would not increase [13]. Hence, we performed general anesthesia using inhaled anesthetics and there were no specific events during anesthesia. However, further studies are needed to clarify the association between PTLS and MH susceptibility.

In summary, prior to anesthetic management, anesthesiologists need to be aware of the variable phenotype of PTLS and the clinical presentation of the patient. As several malformations may be present, preanesthetic evaluation is important and should include echocardiography and cardiologic consultation. It is desirable to prepare for the risk of difficult airway and pulmonary aspiration. The complete recovery of the neuromuscular blockade at the end of the surgery and cautious postoperative care are recommended.

## Data Availability

The data used to support the findings of this study are available from the corresponding author upon request.
